# Mediation-Moderation Links Between Mothers' ACEs, Mothers' and Children's Psychopathology Symptoms, and Maternal Mentalization During COVID-19

**DOI:** 10.3389/fpsyt.2022.837423

**Published:** 2022-03-17

**Authors:** Daphna G. Dollberg, Keren Hanetz-Gamliel

**Affiliations:** School of Behavioral Sciences, Academic College of Tel Aviv-Yaffo, Tel Aviv-Yafo, Israel

**Keywords:** maternal ACEs, maternal anxiety, maternal depression, maternal mentalization, mind-mindedness, children's behavior problems, COVID-19

## Abstract

Research has suggested adverse childhood experiences (ACEs) as a transdiagnostic risk factor for a variety of affective disorders. They are also linked with a parent's tendency toward affect dysregulation and hyperarousal, which may interfere with parenting and children's wellbeing. On the other hand, maternal mentalization can serve as a moderating factor that can help parents regulate their arousal, shielding children during adverse circumstances. We studied the mediated links between ACEs and mothers' and children's psychopathology symptoms during COVID-19 to determine whether maternal mentalization and the child's age moderate these links. Using results from 152 Israeli mothers of children aged 3–12 years recruited during the month-long lockdown in Israel, we documented that the mothers' ACEs were linked with increased risk of depressive and anxiety symptoms and with children's internalizing and externalizing behaviors. Moreover, as hypothesized, the mothers' symptoms of depression and anxiety mediated the links between their ACEs and their children's internalizing behaviors. In addition, the mothers' mentalization skills and, in the case of their depressive symptoms, their child's age, moderated these indirect links. For mothers of young children (3–6 years old) with higher mentalization levels, the link between the mothers' ACEs and the children's behavior problems was weaker compared to mothers with low mentalization levels. For mothers of older children (6–12 years old), and only in the case of maternal depressive symptoms, higher levels of maternal mentalization were linked with more internalizing behaviors. We discuss the potential clinical implications of the findings.

## Introduction

Adverse childhood experiences (ACEs) consist of emotional, physical, and sexual abuse, neglect, household dysfunction and other forms of trauma to which people are exposed before age 18. Building on the first large-scale ACE study in the US by Felitti et al. (1), research has shown that growing up in a dysfunctional household, being exposed to maltreatment or domestic violence or losing a parent through divorce, separation or death may simultaneously affect the nervous, immune and endocrine/metabolic systems, increasing one's risk of poorer physical health outcomes (1–4) and a host of mental health and socioemotional difficulties (4–8). These findings are evident in low-risk community samples worldwide [see (4, 8) for reviews] as well as high-risk samples [e.g., (2)]. The ACE framework suggests that adverse childhood experiences tend to correlate and have a dose-response relationship. Thus, cumulative levels of adversity produce graded decrements throughout development and across domains (4).

Researchers suggest viewing ACEs as a transdiagnostic risk factor for various forms of psychopathology, particularly affective disorders (7). Using the perceptual control theory, Hoppen and Chalder (7) proposed a theoretical model according to which exposure to ACEs early in life may lead to the dysregulation of the autonomic nervous system and the hyper-reactivity of the hypothalamic-pituitary-adrenal axis. These disruptions, in turn, interfere with the operation of the stress response and immune systems, increasing one's risk for physical and emotional diseases (5). Moreover, because these disruptions occur in childhood when the brain is growing rapidly, they interfere with developmental processes and may consequently compromise long term adaptation, leading to socioemotional difficulties. Less is known about psychological resilience in the context of ACEs (7).

Another limitation of ACE research is the fact that the majority of studies have been conducted in the US, Western Europe, and other English-speaking countries, with only limited data available from non-Western countries [see (8–12)]. To the best of our knowledge, research involving ACEs is relatively rare in Israel [see (12, 13), as exceptions].

Developmental research has documented links between ACEs and mothers' mental health difficulties, particularly depression and stress (6, 14, 15), and to a lesser degree, anxiety (6). The mothers' mental health difficulties were then linked with their children's outcomes. Maternal depression as well as attachment insecurity mediated the link between ACEs and the internalizing and externalizing problems of 5-year-old children. However, there were no similar links between maternal anxiety and the children's behavior problems (16). These findings are attributed to the mothers' painful memories related to their ACEs, which may foreshadow their parenting skills (15, 17–19). The ineffective parenting, as well as the mothers' affect dysregulation (20), may interfere with the children's ability to develop appropriate emotion regulation strategies for coping with stress, which may lead to them developing behavior problems (16). Given that the majority of studies on ACEs, parenting and children's outcomes have focused on young children, we do not know whether the same applies to older children.

The outbreak of the COVID-19 pandemic has led to a worldwide increase in mental health problems [e.g., (21–23)], particularly a rise in depressive and anxiety symptoms, with women with children reporting the highest level of symptoms (24). Reports worldwide have documented an increase in children's emotional and behavioral problems (25, 26) such as fears, inattention, irritability, regression and clinginess (27), as well as difficulties in concentration, restlessness, nervousness and worries (28). The threat of the disease, its negative health and economic consequences, and the lockdowns and social distancing regulations have all been implicated as responsible for the rise in psychopathology (29–31). A large national survey conducted in Israel shortly after the pandemic's outbreak and following the first lockdown (32) found that 22.5% of surveyed adults (aged 21 and above) reported an increase in mental health difficulties, particularly depressive, anxiety, and stress symptoms. Additionally, 25.8% of the responders said that their children's emotional state had worsened since the COVID-19 outbreak. Studies have shown that parents' depression and anxiety symptoms during the pandemic accounted for these increases in children's behavior problems, particularly their internalizing behaviors (33). We also found higher levels of Israeli mothers' anxiety symptoms and preschoolers' internalizing and externalizing problems when comparing pre-COVID-19 and COVID-19 samples (34).

As noted above, being exposed to childhood adversity is longitudinally linked with emotional dysregulation and an overburdened stress response system, which may interfere with one's ability to cope with life's challenges. Therefore, we would expect those who have endured more ACEs to have more emotional difficulty dealing with the pandemic than their counterparts who have not experienced such adversity. Indeed, recent studies documented more depressive symptoms (35, 36), anxiety, PTSD (37), and peritraumatic distress (38) following the pandemic's outbreak among individuals who reported more ACEs. The impact of ACEs was both direct (39) and indirect. There was a greater incidence of negative pandemic-related events such as economic hardship and disrupted family functioning among those who had endured many ACEs, which, in turn, were associated with significant increases in symptoms of stress and depression (38).

COVID-19 has been particularly taxing for mothers who experienced more ACEs, with many reporting symptoms of distress, anxiety and PTSD (40). Srivastav et al. (39) argued that, given the evidence of the intergenerational transmission of trauma and the increased risk of ACEs for children of parents who had experienced ACEs, the stressors associated with COVID-19 may create further exposure to ACEs for the children. In sum, we argue that the cumulative negative impact of COVID-19 and ACEs may jeopardize the mother's wellbeing and may also spill over onto the children, jeopardizing their wellbeing as well. However, we also seek to identify protective factors for these children and mothers.

### Maternal Mentalization

We maintain that mothers' mentalization is a potential protective factor in the context of ACEs and the pandemic. Mentalization refers to the quintessential human ability to understand one's self and others in terms of intentional mental states such as feelings, desires, wishes, attitudes and goals. The ability to mentalize effectively is considered a transdiagnostic and transtheoretical capability that is implicated in a wide range of psychological problems and disorders. Specifically, poor mentalization is suggested as a core transdiagnostic element in various forms of psychiatric psychopathologies, including affective, thought, and personality disorders (41).

Parental mentalization is a domain-specific aspect of mentalization. It is defined as a parent's tendency to treat his/her child as an individual with an independent mind that is separate from that of the parent (42), to see the child as motivated by internal mental states such as desires and feelings (43), and to reflect on the parent's own internal mental states and how they are shaped and changed by interactions with the child (44). Mothers' mentalization promotes their children's healthy socioemotional development (45) and self-regulation capacities (46). It has also been linked with fewer internalizing and externalizing behaviors among preschoolers (47, 48).

Stress, arousal and trauma may interfere with the parent's ability to mentalize about the child's inner experiences (49). Mothers who experienced hyperarousal related to birth trauma were less accurate in reading their toddlers' mental states and experienced more parental stress (50). Mothers of infants and toddlers with early regulatory disorders reported more parental stress, which was followed by prementalizing, a form of limited, rigid, ineffective parental mentalization (51). However, the links between trauma, stress and parental mentalization are bidirectional. Whereas greater stress and arousal compromise one's ability to mentalize effectively, better mentalization skills can mitigate the negative impact of high levels of arousal (52) and trauma (53). Moreover, the parents' ability to mentalize reflectively about their own attachment experiences during childhood and their ability to recall painful adverse attachment experiences coherently can help break the intergenerational transmission of relational trauma (54).

In support of parental mentalization being a moderator, parents' mentalization skills moderated the association between parental warmth and control and adolescent outcomes (55). Furthermore, parental mentalization helped mothers modulate their own arousal in the face of a simulated baby cry (56) and contain their over-controlling behaviors (57). In a study from our own lab, mothers' mentalization skills moderated the link between the parents' anxiety symptoms and their children's externalizing behaviors (58). Moreover, comparing mothers' reports about their preschoolers' behavior problems before and during COVID-19, we showed that mothers' mentalization moderated the association between the pandemic and the children's externalizing behaviors. Specifically, when mothers had better mentalization skills, the indirect effect of anxiety on the link between COVID-19 and the children's externalizing behaviors was weaker compared to when mothers demonstrated poorer mentalization skills (34).

Most of the research concerning parental mentalization has focused on young children. Little is known about the contribution of maternal mentalization to children's outcomes past their early years. A few exceptions exist. For example, Borelli and her colleagues (57) looked at maternal mentalization in the context of school-aged children. Benbassat and Priel (55) assessed the contribution of parental reflective functioning to adolescents' socioemotional functioning. Interestingly, the authors found both beneficial and negative links between parents' mentalization and adolescents' outcome. Specifically, paternal reflective functioning had a positive association with adolescents' internalizing behaviors and less positive self-perceptions.

### The Current Study

Theory and empirical evidence support the view of ACEs as a transdiagnostic risk factor for affective disorders. On the other hand, parental mentalization is conceptually and empirically linked with the ability to regulate arousal within one's self and in the child. Building on these two approaches, we propose a conceptual mediation-moderation model to examine the links between mothers' ACEs, mental health difficulties, maternal mentalization skills, and children's behavior.

We tested our model during the outbreak of COVID-19, a challenging time for mothers and children, particularly those mothers who experienced more ACEs while growing up. Given the scarce data regarding the links between mothers' ACEs, mentalization and children's outcomes beyond early childhood, we were interested in studying whether these links differed for young children under age six and older children (ages 6–12). Finally, given that trauma tends to be transmitted intergenerationally, we also investigated the contribution of the mother's ACEs to the child's socioemotional adaptation. Therefore, we examined our research hypotheses while controlling for the child's known traumatic experiences.

### Hypotheses

Based on the literature, we hypothesized that:

H1: Mothers' ACEs will be associated with higher rates of depressive and anxiety symptoms in the mothers and more internalizing and externalizing problems in their children.

H2: Maternal depressive and anxiety symptoms will each mediate the links between the mothers' ACEs and their children's internalizing (H2a) and externalizing (H2b) problems over and above the children's own traumatic experiences. Maternal mentalization and the child's age will moderate these associations. Specifically, the mediated links between ACEs, and psychopathological symptoms in the mothers and children will be weaker when maternal mentalization is higher. We will also investigate the direction of the child's age in moderating this effect.

## Materials and Methods

### Participants

Two hundred and sixty-two Israeli mothers of children between the ages of 3 and 12 years old volunteered for the study. Of the 262, 152 mothers completed all of the study's questionnaires and comprised the final sample. Independent sample t-tests and chi-squared tests found no significant differences between those who completed the questionnaires entirely and those who did not in their main demographic variables such as age, education, income and household composition. The mothers' mean age was 38.97 (*SD*_age_ = 5.84), with 59.1% of them having a post-secondary education. A majority of the mothers (92.8%) were cohabiting with a spouse. Many mothers (91%) indicated that they were in good or very good physical health before COVID-19, and none reported being sick with COVID-19 prior to or during the time of the study. The great majority of mothers indicated above average (57.2%) and average (40.1%) pre-COVID-19 family income according to Israeli standards. The children's age ranged from 3 to 12.66 years old (*M*_age_ = 7.08, *SD*_age_ = 2.54). Girls comprised 52% of the sample. All children attended public schools and 95.4% were described by their mothers as being in good health.

Table 1 lists the number of ACEs the mothers endured in childhood. As the table indicates, more than half of the mothers reported experiencing at least one ACE. Moreover, 14.47% of the mothers noted experiencing a significant number of ACEs (four or more). In descending order of frequency, the ACEs included emotional abuse and neglect, sexual harassment, being separated from a parent due to divorce, death, or desertion, physical abuse, living with a depressed or mentally ill family member, and witnessing family violence and substance abuse.

**Table 1:** Prevalence of ACEs among mothers and mothers' reports of their children's traumatic experiences (*N* = 152).

	***n***	**%**
**Mothers' ACEs**
1. Emotional abuse	42	27.6
2. Physical abuse	26	17.1
3. Sexual harassment	32	21.1
4. Emotional neglect	41	27.0
5. Physical neglect	4	2.6
6. Parental separation, divorce or death	32	21.1
7. Mother treated violently	10	6.6
8. Household substance abuse	9	5.9
9. Household mental illness	20	13.2
10. Incarcerated household member	2	1.3
**ACE score**		
0	65	42.8
1	29	19.1
2	24	15.8
3	12	7.9
4 or more	22	14.5
**Children's traumatic experiences**		
1. Death of a close relative	23	15.1
2. Separation from a parent for more than a month	7	4.6
3. Experiencing a life-threatening event	4	2.6
4. Living under the threat of frequent missile attacks	3	2.0
5. Being physically or sexually abused by an adult	1	0.7
**Number of traumatic experiences**		
0	111	73
1	36	23.7
2	1	0.7
3	2	1.3

The majority of mothers reported that their children experienced no traumatic events. When they occurred, the traumatic events included the death of a close relative, separation from a parent for more than a month, experiencing a life-threatening event, living under the threat of frequent missile attacks, and being physically or sexually abused by an adult.

### Procedure

We recruited our sample in the midst of the first outbreak of COVID-19 during the enforcement of a quarantine in Israel (from mid-March until the end of April 2020). During this time, we posted advertisements on parents' social networks and the link to the questionnaire was spread through the snowball technique. Mothers were asked to consider only one of their children in the specified age range when completing the questionnaires. Data collection ended when the quarantine ended, and the school system reopened under certain restrictions. The Academic College of Tel Aviv Yaffo's Ethics Committee approved the study (ref. 2020079). All participants signed an informed consent prior to participation. Participation was anonymous and voluntary. There was no reward or compensation for participation.

### Measures

#### Mothers' Anxiety and Depression Symptomatology

We used the anxiety and depression subscales of the Brief Symptom Index (BSI) (59) to assess the mothers' reported anxiety symptoms and depressive symptoms. The BSI is a widely used, psychometrically validated, reliable self-report questionnaire (60) comprised of 53 items that measure distress. Respondents were asked to answer questions such as: “Over the last month, to what extent did you feel fearful?” on a 5-point Likert-type scale, ranging from 0 (*not at all*) to 4 (*extremely*). Higher scores reflect greater psychopathology. Internal consistency of the anxiety subscale (Cronbach's alpha = 0.78) and depression subscale (Cronbach's alpha = 0.80) in the current study were good.

#### Mother's Adverse Childhood Experiences

We used the Adverse Childhood Experiences Questionnaire (ACE) (61). The ACE consists of 10 yes/no questions related to the respondents' first 18 years of life. “Yes” answers receive 1 point and “No” answers receive 0 points, yielding a total score ranging from 0 to 10. Higher scores reflect more adverse experiences. The questionnaire items are categorized into three groups: abuse (including emotional, physical and sexual abuse), neglect (including emotional and physical neglect), and household dysfunction (including substance use, mental illness, parental separation or divorce, mother being treated violently, and having an incarcerated household member). Cronbach's alpha for this sample was 0.69.

#### Maternal Mentalization

We utilized Meins and Fernyhough (62) mind-mindedness representational measure to assess the mothers' mentalizing abilities. The mothers were asked to provide a description of their child, from which their representational mentalizing abilities could be inferred. This procedure has been used in many studies and has been shown to be reliable and valid (63). In the current study, the mothers were asked to write a description of their child without further guidance. Coding consisted of identifying comments that included internal-state terms referring to the child's mind such as references to the child's wishes, desires, mental states (e.g., thoughts, knowledge, interests), mental processes (e.g., recognition, remembering, decision making), emotions and attempts to manipulate people's beliefs (e.g., joking, teasing). A mother's mind-mindedness score was determined by the rate of mental-related statements out of the total statements she provided when describing her child. Higher rates indicate a higher mentalizing level.

Two graduate students were trained by the first author, who herself was trained by Prof. Meins, on administering, coding and interpreting the mind-mindedness procedure, on coding the transcripts. Precoding reliability was established by coding training interviews and reaching a level of at least 80% agreement with the expert coder on what constituted mental-related vs. non-mental-related statements. During the coding, we tested interrater agreement on 20 randomly selected interviews (consisting of 213 statements) and determined that the rate was very good (*kappa* = 0.94).

#### Children's Behavior Problems

We measured this factor using the parental Child Behavior Checklist questionnaire. This questionnaire has two versions, depending on the child's age. For children 3–5, mothers completed the CBCL 11/5- 5-years-old (64), and for children 6 and older, they completed the CBCL 6–18 years old (65). In both versions, items are presented on a 3-point Likert-type scale, ranging from 0 (*not true*), through 1 (*somewhat or sometimes true*), to 2 (*very or often true*). The CBCL for preschoolers contains 99 items. The CBCL for school age children contains 113 items. The two versions yield two clusters: internalizing behavior and externalizing behavior. In our study, both clusters demonstrated good internal consistency (for younger children: internalizing behavior: Cronbach's alpha = 0.88; externalizing behavior: Cronbach's alpha = 0.90; for older children: internalizing behavior: Cronbach's alpha = 0.83, externalizing behavior: Cronbach's alpha = 0.91). The CBCL has standardized scores with Israeli norms for each age category (66). There were no significant correlations between the children's age and the raw scores of their internalizing or externalizing behavior in any age group. Thus, we converted the raw scores to t-scores for each age group and used them across the two age groups.

#### Child's Exposure to Traumatic Events

We created a brief questionnaire consisting of six items derived from the Traumatic Events Screening Inventory- Parent Report Revised (TESI-C-PPR) (67) to assess the degree to which the child was exposed to traumatic events. Example items included “experiencing the death of a very close relative,” “separation from a parent for more than a month,” “experiencing a life-threatening event,” and “being physically or sexually abused by an adult.” We added an item about experiencing repeated, prolonged missile attacks. In addition to answering yes or no to each question, we asked the mothers to expand on their responses. “Yes” answers received 1 point and “No” answers received 0 points, yielding a total score ranging from 0 to 6 with higher scores reflecting more traumatic experiences.

#### Demographic and COVID-19-Related Information

Mothers were asked to report basic demographic data about themselves such as age, education and family income. We also measured the mothers' subjective perceptions about the threat of the pandemic using a 3-item questionnaire designed specifically for our study. The questions addressed the mothers' assessment of the long-term implications of the pandemic for their physical and mental health, and the family's economic situation on a 5-point Likert-type scale, ranging from 1 (*destructive implication*) to 5 (*no implication*). The mean score was reversed to correspond to the other variables in the study, so that a higher score indicated more perceived threats. To assess the mothers' sense of being supported, we asked two questions: “To what extent do you feel you received support from extended family members during the quarantine?” and “To what extent do you feel you received support from friends or neighbors during the quarantine?” that were rated on a 3-point scale ranging from 1 (*very little support*) to 3 (*a lot of support*). The mean score was reverse coded to reflect a lack of social support and to correspond to the other variables in the study, so that a high score reflected a sense of lack of social support.

### Data Analysis

We analyzed the data using IBM SPSS Statistics version 27 and the PROCESS macro version 4.0 for SPSS (68). Prior to testing the study's hypotheses, we conducted preliminary analyses considering associations between various demographic factors and the study's variables to identify possible confounders. We conducted a power analysis to determine that the sample's size was sufficient to test our models. We also examined the descriptives of the COVID-19 and ACE related data provided by the respondents. To test H1, we computed the zero-order associations between the ACEs and the mothers' depression and anxiety symptoms and their children's internalizing and externalizing behaviors. Given the different nature of the scales, we used Spearman's rho to examine the correlations with the ACEs, and Pearson's r to examine those between the mothers' depression and anxiety symptoms and their children's internalizing and externalizing behaviors. To test H2a and H2b, we used PROCESS model 72 to investigate the mediating and moderating effects with 5,000 bias-corrected bootstrap samples (68). PROCESS allowed us to combine mediation and moderation within a single model and estimate the conditional and unconditional direct and indirect effects. We ran the analyses separately for the child's internalizing (H2a) and externalizing (H2b) behaviors and for maternal anxiety and depressive symptoms, resulting in four models. In each model we tested the direct and indirect links between ACEs and the child's behaviors (externalizing or internalizing) mediated by the mothers' psychopathology symptoms (anxiety or depression) and moderated by their mind-mindedness and the child's age group over and above the child's traumatic experiences. We considered the effects significant at *p* < 0.05. When the 95% confidence interval included 0, we inferred a significant indirect effect at the 0.05 level. Given the relatively small sample size, we applied the bias-corrected bootstrap samples method (69).

## Results

Preliminary examination showed that all of the study's variables were normally distributed. Examining the zero-order Pearson correlations and chi-squared tests between the sample's demographics and the study's variables revealed no significant associations. The only exception was a significant positive association between the child's age and internalizing behaviors (r = 0.17, *p* < 0.05), with mothers of older children reported more internalizing behaviors. Table 2 shows the means and SDs of the study's variables and the intercorrelations between them, as well as the associations between the study's variables and the mothers' COVID-19 related reports. As the table indicates, the mothers' reports of concerns about COVID-19's implications were significantly and positively associated with their reports of anxiety and depressive symptoms in themselves. Moreover, mothers who reported more such symptoms also reported lacking social support during the lockdown. Reports of COVID-19 concerns also had a positive association with mothers' reports of their children's externalizing behaviors. However, reports of COVID-19 concerns and lack of support were not associated with the mothers' ACEs.

**Table 2:** Descriptive statistics of the study's variables and their intercorrelations (*N* = 152).

	**M/** **SUM**	**SD**	**Threat of COVID-19**	**Lack of social support**	**Child's traumatic experiences**	**Mother's** **ACEs**	**Mothers' anxiety**	**Mothers' depression**	**Mother's** **MM**	**Child's externalizing** **behavior**
Threat of COVID-19	3.76	7.24								
Lack of social support	2.01	0.68	0.07							
Child's traumatic experiences	0.29	0.55	0.00	0.14						
Mother's ACEs	1.44	1.73	−0.03	−0.08	0.017					.
Mothers' anxiety	0.90	0.51	0.31^**^	0.18^*^	−0.03	0.19^**^				
Mothers' depression	0.70	0.54	0.28^**^	0.19^*^	−0.05	0.28^**^	0.71^**^			
Mother's MM	0.61	0.25	0.06	0.05	0.09	0.10	0.04	0.04		
Child's externalizing behavior	49.87	9.27	−0.12	−0.15	0.07	0.09	0.25^**^	0.22^**^	0.007	
Child's internalizing behavior	50.87	9.75	−0.18^*^	−0.03	0.11	0.19^*^	0.34^**^	0.31^**^	0.01	0.58^**^

* *p <0.05,*

** *p < 0.01. MM, Mind-mindedness*.

To test H1 we examined the zero-order correlations among the study's variables (see Table 2). We found that, as hypothesized, the mothers' ACEs scores correlated positively with their reports of depressive and anxiety symptoms. These scores also correlated significantly and positively with their reports of their children's internalizing behavior. However, contrary to our prediction, there was no significant association between the mothers' ACEs scores and their children's externalizing behavior. Nevertheless, the mothers' reports of their anxiety and depressive symptoms correlated positively with their children's internalizing and externalizing behavior. Thus, higher levels of maternal psychopathology were associated with more internalizing and externalizing behaviors in their children. Note that the mothers' mind-mindedness was not correlated with any of the study's variables. Furthermore, there was a strong positive association between depression and anxiety symptoms, indicating a high level of comorbidity. In sum, H1 was generally supported.

To test H2 regarding the mediating and moderating links between the mothers' ACEs, psychopathology symptoms, mind-mindedness, the children's age, and their internalizing (H2a) and externalizing (H2b) behaviors while controlling for their traumatic experiences, we tested four regression models. A power analysis using G^*^Power3.1.9.2 software showed that the sample size was sufficient for detecting a medium-sized (f^2^ = 0.35) mediation-moderation effect with a probability of 99.8%. As hypothesized, the model predicting the children's internalizing behavior from ACEs, mediated by their mothers' depressive symptoms and moderated by their mind-mindedness and the children's age over and above the child's traumatic experiences, was significant [*F*_(9, 140)_ = 3.37, *p* < 0.001 R^2^ = 0.18]. We found a significant three-way interaction [*F*_(1, 140)_ = 4.85, *p* < 0.05, R^2^ change = 0.03]. Thus, the mothers' depressive symptoms mediated the association between their ACEs and their children's internalizing behavior. This link varied depending on the mother's mind-mindedness and the child's age. The direct link between ACEs and the children's internalizing behavior was insignificant. We tested the indirect links at three levels of mind-mindedness (low = 1SD below average, medium = average, and high = 1 SD above average) and for the two age groups (young = below 6 years old, older = 6–12 years old). Table 3 lists the results.

**Table 3:** Indirect links between the mother's ACEs, her psychopathology symptoms, and her child's behavior moderated by the mother's mind-mindedness and the child's age.

		**Mother's depression**			**Mother's anxiety**		
		**B**	**SE**	**LLCI, ULCI**	**B**	**SE**	**LLCI, ULCI**
**Mother's ACEs** ** → child's internalizing behavior**							
Young children	Low level of mother's MM	1.33^*^	0.69	0.06, 2.85	1.31^*^	0.68	0.10, 2.74
	Medium level of mother's MM	0.96^*^	0.47	0.03, 1.90	0.42	0.46	−0.43, 1.42
	High level of mother's MM	0.60	0.85	−1.70, 1.85	−0.25	0.70	−1.98, 0.87
Older children	Low level of mother's MM	−0.04	0.11	−0.24, 0.23	0.02	0.10	−0.11, 0.30
	Medium level of mother's MM	0.13	0.13	−0.06, 0.47	0.16	0.14	−0.02, 0.51
	High level of mother's MM	0.66^*^	0.35	0.11, 1.50	0.43	0.37	−0.13, 1.30
**Mother's ACEs** ** → child's externalizing behavior**							
Young children	Low level of mother's MM	0.97^*^	0.67	0.14, 2.76	1.00^*^	0.53	0.18, 2.22
	Medium level of mother's MM	0.76^*^	0.44	0.06, 1.75	0.29	0.33	−0.28, 1.05
	High level of mother's MM	0.57	0.61	−0.60, 1.89	−0.16	0.43	−1.01, 0.76
Older children	Low level of mother's MM	0.02	0.13	−0.29, 0.28	0.07	0.14	−0.11, 0.46
	Medium level of mother's MM	0.17	0.16	−0.16, 0.50	0.19	0.15	−0.05, 0.55
	High level of mother's MM	0.46	0.32	−0.11, 1.15	0.35	0.31	−0.15, 1.07

* *p < 0.05.*

*MM, Mind-mindedness; Young children = 3–6 years old. Older children = 6–12 years old*.

In the younger group, the mother's depressive symptoms mediated the link between ACEs and the children's internalizing behavior only when the mother's mind-mindedness was low or average. However, for the older children, the mother's depressive symptoms mediated this link when the mother's mind-mindedness was high. Specifically, ACEs had the strongest effect on the internalizing behavior of young children of mothers with low levels of mind-mindedness {B = 8.42, SE = 2.46, *t*_(150)_ = 3.42, *p* < 0.001 [3.56, 13.29]}. A weaker though still significant positive effect was also evident in young children of mothers with average levels of mind-mindedness {B = 6.25, SE = 1.98, *t*_(150)_ = 3.15, *p* < 0.001 [2.33, 10.17]}. In contrast, when the mother's level of mind-mindedness was high and the child was young, there was no association between the mother's ACEs and the child's internalizing behavior.

A reverse pattern emerged for the older children. There was a significant link between ACEs and the children's internalizing behavior when the mother's level of mind-mindedness was high (B= 6.96, SE = 6.96, *p* = 0.01 [1.87, 12.04]), whereas when it was average or low, the links were insignificant. Pairwise contrasts showed that the difference between the indirect effects based on the mother's mind-mindedness level and the child's age were significant. These effects were evident over and above the child's own traumatic experiences, which were unrelated to their internalizing behavior.

The model predicting the children's internalizing behavior from ACEs, mediated by the mothers' anxiety symptoms and moderated by their mind-mindedness and the child's age, while controlling for the child's traumatic experiences, was also significant [*F*_(9, 140)_ = 3.67, *p* < 0.001, R^2^ = 0.19]. There was a three-way interaction between ACEs, the mother's mind-mindedness and the child's age predicting the mother's anxiety symptoms [*F*_(1, 141)_ = 3.94, *P* = 0.05, R^2^ change = 0.02]. Thus, the mother's ACEs predicted her anxiety symptoms only when she had little mind-mindedness and the child was young {B = 0.14, SE = 0.05, *t*_(150)_ = 2.71, *p* = 0.01 [0.04, 0.25]}. Moreover, as Table 3 indicates, in such situations, the mother's anxiety symptoms mediated the links between her ACEs and the child's internalizing behaviors. Pairwise contrasts showed that the difference between the indirect effects based on the mother's mind-mindedness level and the child's age was significant. The direct link between ACEs and the child's internalizing behavior was insignificant. In sum, H2a was mostly supported.

The model predicting the child's externalizing behavior from ACEs, mediated by the mother's depressive symptoms and moderated by mind-mindedness and the child's age, was only marginally significant [*F*_(9, 140)_ = 1.84, *p* = 0.07, R^2^ = 0.11]. However, only the child's age had a marginally significant direct effect on the child's externalizing behavior, such that older children exhibited more externalizing behaviors than younger children {B = 13.24, SE = 7.06, *t*_(150)_ = 1.87, *p* = 0.06 [−0.73, 27.20]}.

The model predicting the child's externalizing behavior from ACEs, mediated by the mother's anxiety symptoms, and moderated by her mind-mindedness and the child's age, was significant [*F*_(9, 140)_ = 1.84, *p* < 0.05, R^2^ = 0.12]. There was a significant three-way interaction of ACEs by mind-mindedness and the child's age and the mother's anxiety symptoms [*F*_(1, 141)_ = 3.94, *p* = 0.05, R^2^ change = 0.02]. Specifically, for young children, when the mother's mind-mindedness was low, the effect of ACEs on the mother's anxiety symptoms was significant and positive {B = 0.14, SE = 0.02, *t*_(150)_ = 2.71, *p* = 0.01 [0.04, 0.25]}, indicating that more ACEs were associated with more anxiety symptoms. No similar effect emerged when the mothers had average or high levels of mind-mindedness or in the older age group. Finally, there was a significant indirect link between ACEs and the externalizing behavior of young children whose mothers demonstrated low levels of mind-mindedness. However, a pairwise contrast analysis revealed that the effect was not significantly different from the other indirect effects. No significant direct link was found either. Thus, H2b was rejected.

## Discussion

We conducted our study during the initial surge in COVID-19 in Israel, when the country was under lockdown, families were confined to their homes, their daily routines were disrupted and they were isolated from their usual social support networks. These circumstances created great stress and were likely accompanied by affect dysregulation for mothers and children.

Supporting H1, we found that mothers who had experienced more ACEs also reported more depressive and anxiety symptoms and their children showed more internalizing behaviors. However, we found no simple associations between ACEs and children's externalizing behaviors. Supporting H2a, the links between the mother's ACEs and her child's internalizing behaviors were mediated by her symptoms of anxiety and depression. Moreover, as predicted, these links were moderated by the mother's mentalization skills and, in the case of the mother's depressive symptoms, by the child's age as well. Specifically, having experienced more ACEs was linked with more maternal depressive and anxiety symptomatology and with more internalizing behavior in their children. This effect occurred only in young children of mothers with poor mentalization skills. In contrast, as predicted, when the mother's mentalization was stronger, no such llinks were evident. Among older children and only with regard to maternal depressive symptoms, mothers who experienced more ACEs reported more depressive symptomatology and more internalizing behaviors in their children. These links were evident only in mothers with strong mentalization skills whereas when the mother's mentalization level was lower, there were no significant effects.

We rejected H2b because the model predicting children's externalizing behavior via their mothers' depressive symptoms was only marginally significant. Furthermore, we found none of the predicted direct, mediated and moderated links. In addition, the model predicting children's externalizing behavior from ACEs and their mothers' anxiety symptoms was significant. As expected, ACEs predicted the mother's anxiety symptoms when her mentalization level was low and the child was younger. However, the hypothesized mediated-moderated link between the mother's anxiety symptoms and the child's externalizing behaviors was not supported.

Our findings regarding the positive association between ACEs and the mothers' anxiety and depressive symptoms are consistent with mounting evidence regarding the negative impact of ACEs on adults' mental health (4–8), particularly following the outbreak of COVID-19 (35–39). Importantly, ACEs were linked with both anxiety and depressive symptomatology, which were strongly correlated, indicating the comorbidity between them. This comorbidity, as well as the similar pathways between ACEs and these two forms of affect dysregulation disorders, support the view of ACEs as a transdiagnostic risk factor for affective disorders. Thus, exposure to adversity in childhood may initiate a negative developmental trajectory that may interfere with the development of a solid, flexible, adaptive system for coping with stress. In the absence of such a system, stressful events encountered later in life may lead to allostatic load, chronic emotional dysregulation and a life-long difficulty in coping with various adult challenges, including parenting and childrearing.

Our findings also join a rapidly growing body of literature indicating that a mother's ACEs can interfere with her child's socioemotional adaptation (70). They suggest that a mother's affective dysregulation, manifested in her anxiety and depressive symptoms, is one possible mediating mechanism between her ACEs and her child's behavior problems. Given what is known about ACEs, it is possible that painful, traumatic childhood memories lead to a unique mental state (18) and affect dysregulation (20), which, in return, undermines the mother's ability to parent effectively. In line with this reasoning, research shows that caregivers who have experienced many ACEs tend to feel that their relationship with their child is going the wrong way, are more critical of their child's behavior and feel they have less control over their lives (12). Similarly, a recent qualitative meta-analysis reported that mothers who experienced childhood adversity reported of constant overwhelming anxiety about their child's safety, a tendency to overprotect the child and hypervigilant parenting. These mothers were preoccupied by the desire to create a childhood for their children that was different than theirs and consequently struggled to find appropriate discipline strategies (71). These concerns and preoccupations, as well as the mothers' depressive and anxiety symptoms, may lead to unhealthy parenting practices and parenting stress in the mothers. They may jeopardize their children's ability to develop appropriate emotion regulation strategies for coping with stress, increasing their risk of developing behavior problems (16).

Therefore, we argue that for mothers who have endured childhood adversity, coping with the pandemic may have created an additional emotional burden, increasing their risk of depression and anxiety. In turn, these anxiety and depressive symptoms may lead these mothers, who are predisposed to becoming stressed and dysregulated, to be more overwhelmed by the short-term and long-term health and social consequences of the pandemic than mothers with no similar mental health problems or a history of ACEs. This speculation is supported by the co-occurrence of high rates of depressive and anxiety symptoms and mothers' concerns regarding the pandemic's implications and lack of social support in our study. Thus, the cumulative stress of ACEs and the current pandemic may exacerbate the mother's negative mood and intensify her negative state of mind with regard to parenting. These factors, separately and together, interfere with the mother's ability to parent her child effectively, accounting for the children's internalizing and externalizing behaviors.

Importantly, and in line with our prediction, maternal mentalization skills moderated the links between the mother's ACEs, her affective symptoms and her child's behavior. Specifically, strong maternal mentalization skills mitigated the links between the mothers' ACEs, their depressive and anxiety symptoms and their children's internalizing behaviors. These skills also mitigated the link between ACEs and the mothers' anxiety symptoms in the case of their children's externalizing behaviors. These findings accord with previous studies demonstrating the potentially protective effect of maternal mentalization [e.g., (54–58)]. It is possible that high levels of maternal mentalization may help mothers regulate their arousal resulting from their ACEs and the pandemic, and recognize and attend to their child's regulation needs, at least among young children. Moreover, higher levels of maternal mentalization may help mothers appreciate their young children's developmental needs, allowing them to differentiate between their children's mental experiences and their own, and recognizing the merit of trying to regulate their negative emotions so as not to overwhelm their young children. Furthermore, the mothers' ability to think about their children's mental state helps the children develop their own self-regulation skills (46). Consequently, their children show fewer internalizing behaviors. In contrast, mothers with fewer mentalization skills are less able to self-modulate their own affect dysregulation and that of their children. They may flood their children with their own worries regarding their safety, which may increase their young children's fears and worries.

In general, the mediated-moderated links were less apparent in older children. Moreover, in this age group, the links between ACEs and the children's internalizing problems were stronger in mothers with stronger mentalization skills. This finding echoes Benbassat and Priel's (55) report of a positive association between paternal reflective functioning and adolescents' internalizing behaviors. This finding is not surprising given the relatively higher rates of internalizing symptoms among the older children found in our study as well as in other studies conducted following the COVID-19 outbreak [e.g., (72, 73)]. Taken together, we argue that during later stages of development, and particularly during a stressful time such as the current pandemic, mothers with better mentalization skills may be more attuned to their older children's signs of distress, despite their own distress and history of traumatic experiences. They may recognize boredom, lack of motivation, daytime sleeping, and screen addiction in their adolescents as evidence of this distress. In contrast, mothers with fewer mentalization skills who have experienced many ACEs may be over-focused on their own stress and needs, and may miss the older children's signs of distress.

Of note is the fact that in our study, the links between ACEs and maternal psychopathology symptoms were mostly evident in the case of children's internalizing, as opposed to externalizing, behavior. This result may reflect the worldwide rise in children's fears, worries, anxiety and depression following the outbreak of COVID-19 (25–28). It is also possible that mothers who have experienced many ACEs, and therefore tend to be more anxious and depressed (which are considered as internalizing symptoms), may project their own fears, concerns and negative affect onto their children. They may also unconsciously reinforce their children's internalizing difficulties by overanalyzing their behavior, looking for signs of distress and by being overprotective of them (71).

### Contribution, Limitations, and Future Directions

Our study adds to and strengthens existing evidence regarding the contribution of ACEs to the risk of mothers' and children's psychopathology, and the moderating role of mentalization as a regulatory mechanism in the face of arousal and affect dysregulation. Its added contribution lies in expanding the discussion of the impact of ACEs not only on adults but also on their offspring and not only during the early stages of child development but also into early adolescence. It also expands the existing research by testing ACE models during COVID-19, supporting the claim that the pandemic has affected people differently based on their preexisting fragility and resilience. Importantly, our study involved low-risk, high-functioning mothers. Nevertheless, more than half of the mothers in the sample reported at least one adverse childhood experience and almost 15% reported four or more ACEs, supporting the argument that ACEs are quite prevalent across communities and nations. Moreover, a few of the children were also reported to have experienced a traumatic event firsthand. However, we established that the links between the mothers' ACEs and their children's behavior were over and above the children's own traumatic experiences. This finding adds to the large body of research highlighting the important contribution, positive and negative, of parents' childhood histories to their children's wellbeing.

Nevertheless, several caveats need to be mentioned. The homogeneity of the sample of low-risk, high-functioning, cohabiting mothers limits the generalizability of the findings to other, more at-risk samples. The concurrent assessment and the retrospective nature of the reports about ACEs also limit our ability to form causal links between childhood adversity, current mental health and children's behavioral outcomes. It is equally possible that the mothers' mental health difficulties, exacerbated by the pandemic, influenced their retrospective accounts of their childhood, portraying it in more negative terms than actual, objective reality. Furthermore, the relatively modest sample size precluded to test additional and more complex hypotheses and may have limited our ability to detect additional significant effects. Finally, all of the measures came from the mothers, which may have created an informer bias. Future studies can benefit from using multiple informants including fathers, teachers and the children themselves. Longitudinal designs that follow children who have been exposed to ACEs into adulthood and parenthood can help us better understand the developmental trajectory associated with ACEs and the conditions under which mentalization skills emerge, fail to develop or collapse. Observational studies looking at parenting practices and the actual utilization of mentalizing skills among parents who endured ACEs can help identify the specific behavioral mechanisms by which ACEs dysregulate parents.

### Clinical Implications

Unprocessed childhood adversity may affect the psychobiology of parenting and may interfere with a mother's wellbeing as well as her child's wellbeing and adaptation. Therefore, offering psychotherapy to survivors of childhood adversity is important. Moreover, our findings show that poor maternal mentalization, when coupled with ACEs, may further increase the risk that mothers will become dysregulated during stressful circumstances such as the current pandemic, which, may increase the risk of young children developing internalizing behavioral problems. The rise in mental health problems in adults and children during the pandemic points to the need to mentally support parents and children at this time. Research and clinical evidence show that highlighting benevolent childhood experiences can neutralize the effects of ACEs (74), particularly during the current pandemic (35). Similarly, Lieberman et al. (75) coined the concept of “angels in the nursery” to suggest that underscoring shared positive affective experiences between the adult as a child and his/her caregiver can promote growth in traumatized parents and is vital to effective psychotherapy with them. Indeed, recent studies have demonstrated the protective effect of positive childhood memories in caregivers against the intergenerational transmission of trauma (76). Thus, mental health professionals who work with mothers who report a history of childhood adversity should focus not only on dealing with these experiences but also encourage their recollection of positive, benevolent memories. Furthermore, several evidence-based interventions that focus on improving parental mentalization are currently available for parents of younger children (77, 78) and older children (49, 79, 80). Improving parental mentalization skills can help dysregulated mothers regulate themselves and better understand their children's needs. Doing so may be especially important when the child's needs are overshadowed by the mother's childhood ghosts (81) and traumatic past experiences (82).

## Data Availability Statement

The raw data supporting the conclusions of this article will be made available by the authors, without undue reservation.

## Ethics Statement

The studies involving human participants were reviewed and approved by the Academic College of Tel Aviv Yaffo's Ethics Committee approval reference #2020079. The patients/participants provided their written informed consent to participate in this study.

## Author Contributions

The manuscript was based on original data collected by the team of DD and KH-G. DD and KH-G were the primary researchers in this project and have co-authored the current submitted manuscript. Both authors equally contributed to the study conception and design and write-up, performed material preparation, data collection, and analysis, and contributed to the article and approved the submitted version.

## Conflict of Interest

The authors declare that the research was conducted in the absence of any commercial or financial relationships that could be construed as a potential conflict of interest.

## Publisher's Note

All claims expressed in this article are solely those of the authors and do not necessarily represent those of their affiliated organizations, or those of the publisher, the editors and the reviewers. Any product that may be evaluated in this article, or claim that may be made by its manufacturer, is not guaranteed or endorsed by the publisher.
